# Pharmacokinetic Evaluation of Neutral Sphinghomyelinase2 (nSMase2) Inhibitor Prodrugs in Mice and Dogs

**DOI:** 10.3390/pharmaceutics17010020

**Published:** 2024-12-26

**Authors:** Arina Ranjit, Chae Bin Lee, Lukáš Tenora, Vijaya Saradhi Mettu, Arindom Pal, Jesse Alt, Barbara S. Slusher, Rana Rais

**Affiliations:** 1 Department of Neurology, Johns Hopkins School of Medicine, Baltimore, MD 21205, USA; 2 Johns Hopkins Drug Discovery, Johns Hopkins School of Medicine, Baltimore, MD 21205, USA; 3 Departments of Psychiatry and Behavioral Sciences, Johns Hopkins School of Medicine, Baltimore, MD 21205, USA; 4 Department of Pharmacology and Molecular Sciences, Johns Hopkins School of Medicine, Baltimore, MD 21205, USA; 5 Department of Oncology, Johns Hopkins School of Medicine, Baltimore, MD 21205, USA; 6 Department of Neuroscience, Johns Hopkins School of Medicine, Baltimore, MD 21205, USA; 7 Department of Medicine, Johns Hopkins School of Medicine, Baltimore, MD 21205, USA

**Keywords:** nSMase2, DPTIP, prodrug, mice pharmacokinetics, dog pharmacokinetics, oral bioavailability

## Abstract

**Background**: Extracellular vesicles (EVs) can carry pathological cargo, contributing to disease progression. The enzyme neutral sphingomyelinase 2 (nSMase2) plays a critical role in EV biogenesis, making it a promising therapeutic target. Our lab previously identified a potent and selective inhibitor of nSMase2, named DPTIP (IC_50_ = 30 nM). Although promising, DPTIP exhibits poor pharmacokinetics (PKs) with a low oral bioavailability (%F < 5), and a short half-life (t_1/2_ ≤ 0.5 h). To address these limitations, we previously developed DPTIP prodrugs by masking its phenolic hydroxyl group, demonstrating improved plasma exposure in mice. Recognizing that species-specific metabolic differences can influence prodrug PK, we expanded our studies to evaluate selected prodrugs in both mice and dogs. **Methods:** The scaleup of selected prodrugs was completed and two additional valine- ester based prodrugs were synthesized. Mice were dosed prodrugs via peroral route (10 mg/kg equivalent). For dog studies DPTIP was dosed via intravenous (1 mg/kg) or peroral route (2 mg/kg) and prodrugs were given peroral at a dose 2 mg/kg DPTIP equivalent. Plasma samples were collected at predetermined points and analyzed using developed LC/MS-MS methods. **Results:** In mice, several of the tested prodrugs showed similar or improved plasma exposures compared to DPTIP. However, in dog studies, the double valine ester prodrug **9**, showed significant improvement with an almost two-fold increase in DPTIP plasma exposure (AUC_0–t_ = 1352 vs. 701 pmol·h/mL), enhancing oral bioavailability from 8.9% to 17.3%. **Conclusions:** These findings identify prodrug **9** as a promising candidate for further evaluation and underscore the critical role of species-specific differences in prodrug PKs.

## 1. Introduction

Extracellular vesicles (EVs) are small membranous globules released by cells that play a crucial role in intracellular communication and the regulation of cellular processes [1,2]. EVs are loaded with proteins, nucleic acids, and bioactive lipids, which influence both the physiological and pathological functions of the recipient cells [3]. Under normal physiological conditions, EVs contribute to crucial cellular functions such as cell proliferation, differentiation [4], haemostasis [5], tissue regeneration [6], bone calcification [7], and synapse formation in neurons [8]. However, under pathological state, EVs can play a role in promoting inflammation and facilitating the spread and exacerbation of diseases [9,10].

One of the pathways responsible for EV biogenesis involves the production of ceramide through the hydrolysis of sphingomyelin (SM) by the enzyme neutral sphingomyelinase 2 (nSMase2) [11]. The nSMase2 enzyme is subcellularly localized in the plasma membrane and Golgi apparatus [12]. It is expressed in various tissues including the brain [13], liver [14], kidney [15], lungs [16], intestinal mucosa [17], and tumors [12] with the highest expression observed in the brain [18]. While transient increases in nSMase2 activity and EV release are part of normal cellular function [19], chronic upregulation was linked to pathological conditions, including neurodegenerative and cardiovascular diseases [20,21], cancer metastasis [22], and the spread of infections [23,24,25,26]. Furthermore, pharmacological inhibition of nSMase2 is shown to alleviate disease pathology in preclinical models, making it a promising therapeutic target [27].

Early attempts to discover nSMase2 inhibitors were primarily focused on bacterial, bovine, or rat enzymes [28,29,30,31]. To cite an example, GW4869, one of the most studied inhibitors, was identified using rat brain nSMase2 and exhibited an IC_50_ of 1 µM [32]. However, its poor solubility and lack of drug-like properties prevented its development. Cambinol, discovered by our lab, was the first compound identified as a nSMase2 inhibitor using human recombinant enzymes, but it also displayed weak potency (IC_50_ = 7 µM) [33]. Previously discovered nSMase2 inhibitors have shown limitations such as low potency, suboptimal physicochemical properties, and inadequate brain penetration. To date, no nSMase2 inhibitors have proven suitable for clinical advancement.

Our laboratory identified 2,6-dimethoxy-4-(5-phenyl-4-(thiophen-2-yl)-1*H*-imidazol-2-yl)-phenol (DPTIP) as the first nM inhibitor of nSMase2, (IC_50_ = 30 nM). DPTIP demonstrated high selectivity for nSMase2 and good metabolic stability. However, DPTIP displayed poor pharmacokinetic (PK) properties, including a short half-life (<30 min), and low oral bioavailability in mice (%F < 5) [34,35]. Given that structural modifications in DPTIP scaffold did not yield improvements [35], we used prodrug strategies to advance DPTIP by optimizing its PK properties by masking its phenolic hydroxyl group [36]. Of these prodrugs, diethyl piperidino-piperidine-conjugated DPTIP exhibited over fourfold higher plasma exposure in mice compared to DPTIP. It also effectively inhibited IL-1β-induced EV release into plasma and demonstrated robust target engagement through the significant inhibition of nSMase2 in a mouse model of acute brain injury [36]. These results prompted us to further evaluate these prodrugs.

Prodrugs often display species-specific metabolic differences primarily due to the variations in the activity of enzymes responsible for their biotransformation. Esterases, for instance, play a key role in the hydrolysis of ester-based prodrugs, and can have markedly different expression levels, substrate specificities, and catalytic efficiencies across species [37,38,39]. Understanding these metabolic differences is essential for predicting drug behavior in humans, selecting the appropriate animal models, and designing prodrugs with optimal activation and PK profiles across species [40,41,42].

Our group has successfully evaluated PK properties of drug candidates across species such as rodents [36,43], dogs [44,45], non-human primates [46], and swine [47] by utilizing prodrug strategies that improve solubility [44], permeability across biological membranes [45], brain penetration [46], tumor targeting [43], and lymphoid targeting [47]. Herein, we report the synthesis, stability, and in vivo PK evaluation of selected DPTIP prodrugs in mice and dogs. Among the DPTIP prodrugs synthesized, the newly designed double valine ester prodrug **9** emerged as a promising candidate, improving oral bioavailability by almost 2-fold. This represents a significant step towards developing a novel DPTIP prodrug that could facilitate clinical translation of this class of inhibitors.

## 2. Materials and Methods

### 2.1. Materials

Dog and human plasma were purchased from Innovative Research, Inc. (Novi, MI, USA). N-Methyl-2-pyrrolidine (NMP) was obtained from Omnisolv Inc. (Vienna, VA, USA). Tween and dimethyl sulfoxide (DMSO) were purchased from Sigma Aldrich (St. Louis, MO, USA). Kolliphor and polyethylene glycol (PEG) were purchased from Sigma Life Sciences (Burlington, MA, USA). All reagents used for synthesis or chromatography separation were purchased from commercial suppliers such as Sigma Aldrich (St. Louis, MO, USA), TCI (Portland, OR, USA), Chem-Impex (Wood Dale, IL, USA), Fluorochem (Old Glossop, UK), and Ambeed (Arlington Heights, IL, USA) and were used without further purification. The solvents used were anhydrous or HPLC grade and were obtained from Sigma Aldrich (St. Louis, MO, USA) or Merck (Rahway, NJ, USA).

### 2.2. Synthesis

All reagents were purchased from commercial suppliers like Sigma Aldrich (St. Louis, MO, USA), TCI (Portland, OR, USA), Chem-Impex (Wood Dale, IL, USA), Fluorochem (Old Glossop, UK), or Ambeed (Arlington Heights, IL, USA) and were used without further purification. All the reactions were conducted in standard dried glassware under inert atmosphere (nitrogen). TLC was performed on silica gel 60 F254-coated aluminum sheets (Merck, Rahway, NJ, USA). Column chromatography was performed on silica gel 60 (0.040–0.063 mm, Fluka) or on Biotage Isolera One Flash Chromatography System using SiliCycle SiliaSep cartridges with silica grade 40–63 µm. NMR spectra were measured on Bruker AVANCE 400. ^1^H NMR were recorded at 400 MHz and signals of DMSO-*d*_6_ (δ 2.50, 3.33) were used for standardization. The chemical shifts are given in δ scale; the coupling constants J are given in Hz. Preparative HPLC purification was performed on an Agilent 1200 series HPLC system equipped with an Agilent G1315D DAD detector using a Phenomenex Luna 5 μm C18 column (21.2 mm × 250 mm, 5 μm). Analytical HPLC was performed on an Agilent 1200 series HPLC system equipped with an Agilent G1315D DAD detector (detection at 220 nm) and an Agilent 6120 quadrupole MS detector using an Agilent Eclipse Plus C18 column (2.1 mm × 50 mm, 3.5 μm) at a flow rate of 1.25 mL/min. All final biologically tested compounds were confirmed to be of >95% purity by the HPLC and NMR methods described above.

The synthesis of DPTIP and its prodrugs **2**–**7** were reported previsouly [25,26] and the scale-up batches for all prodrugs followed identical synthetic procedures. Briefly, DPTIP was prepared in 3 synthetic steps starting with phenyl acetylene and 2-iodothiphene. Sonogashira coupling reaction in the presence of Pd-catalyst and CuI provided 2-(phenylethynyl) thiophene, which was oxidized by potassium permanganate to 1-phenyl-2-(thiophen-2-yl) ethane-1,2-dione. In the final step, diketone reacted with 4-hydroxy-3,5-dimethoxybenzaldehyde and ammonium acetate under acidic conditions (acetic acid) to provided DPTIP as a light purple solid. Prodrugs **2**–**5** and **7** were synthesized by a single step reaction using DPTIP, Hünig’s base (DIPEA), and acyl chlorides (prodrugs **3** and **4**) or respective secondary amines (prodrugs **2**, **5** and **7**) after treatment with triphosgene. Prodrug **6** was obtained by a 2 step process including the alkylation of DPTIP with di-tert-butyl (chloromethyl) phosphate in the presence of base (cesium carbonate) and sodium iodide and following the deprotection of t-Bu groups under acidic conditions (trifluoroacetic acid). Since the synthetic procedures of newly added prodrugs **8** and **9** were not described yet, the detailed synthetic methods are described in the “Supplementary Information” file.

### 2.3. Metabolic Stability Studies

The in vitro metabolic stability analysis of DPTIP prodrugs was performed in the plasma of mice, dogs, and humans, as previously described [36]. Briefly, for plasma stability, 1 mL of plasma was spiked with 10 μM of the respective prodrug followed by incubation in an orbital shaker at 37 °C for 1 h (in triplicate). The samples from each incubation at predetermined time points (0 and 60 min) was quenched with 3× volume of acetonitrile containing the internal standard (IS; losartan: 0.5 μM). The samples were vortex-mixed for 30 s and centrifuged at 10,000× *g* for 10 min at 4 °C. Compound disappearance over time was monitored via a Dionex ultra-high-performance LC system coupled with a Q Exactive Focus orbitrap mass spectrometer (Thermo Fisher Scientific Inc., Waltham, MA, USA). Quantification of the prodrugs was performed in full scan mode (from *m*/*z* 75 to 1125) by comparing *t* = 0 min samples with *t* = 60 min samples. Molecular weight and their corresponding [M+H] ^+^ ions are reported in the table (Table S1).

### 2.4. Pharmacokinetic Studies in Mice

In vivo mice PK studies were carried out as previously described [36]. Briefly, mice PK studies were carried out in *CES1^−/−^* mice according to protocols approved by the Animal Care and Use Committee at Johns Hopkins University. The *C57BL/6 CES1^−/^*^−^ mice were received from the United States Army Medical Research Institute of Chemical Defense, Maryland, and maintained on a 12 h light−dark cycle with ad libitum access to food and water. Prodrugs **3**, **4**, **5**, **6**, **8,** and **9** were administered at a dose of 10 mg/kg equivalent of DPTIP to *CES1^−/−^* mice as a single peroral (PO) dose made fresh in formulation (5% DMSO, 10% tween, and 85% saline *v*/*v*/*v*) prior to dosing. The mice were sacrificed at specified time points (0.25, 0.5, 1, 2, 4, 6, and 8 h) post-drug administration with (*n* = 3) per time point for each prodrug. For the collection of plasma, animals were euthanized with CO_2_, and blood samples were collected in heparinized microtubes by cardiac puncture. The blood samples were centrifuged at 3000× *g* for 10 min at 4 °C, and plasma was removed and stored at −80 °C until LC−MS/MS analysis.

### 2.5. Pharmacokinetic Studies in Dogs

The PK studies in dogs were performed in accordance with the guidelines recommended by Pharmaron (San Diego, CA, USA) Institutional Animal Care and Use Committee. Briefly, male Beagle dogs 9 to 11 kg were obtained from the test facility’s colony of non-naïve animals. The animals were fasted overnight prior to dose administration and fed 4 h after dosing. The body weights were recorded and the animals were dosed with DPTIP, and prodrugs **2**, **3**, **4**, **6**, **7**, **8,** and **9** at 2 mg/kg equivalent of DPTIP as a single preoral dose PO (*n* = 2) with the exception of DPTIP IV dosed at 1 mg/kg (*n* = 1). All drugs were dissolved in the formulations (5% NMP, 5% Kolliphor, 30% PEG, and 60% water *v*/*v*) made freshly prior to dosing. The blood samples were collected via a cephalic vein at specified time points (0.083, 0.25, 0.5, 1, 2, 4, 6, 8, and 24 h) post-drug administration, and transferred into tubes containing sodium fluoride as a stabilizer (10 µL/mL of 500 µg/mL). The tubes were stored on wet ice until processed to plasma by centrifugation (3500× *g* at 4 °C for 10 min) within 30 min of collection. The plasma samples were transferred into individually labeled matrix tubes and stored at nominal −80 °C immediately (within 10 min of separation) until bioanalysis.

### 2.6. Bioanalysis

The quantification of the released DPTIP was performed using our previously published method with minor modifications [36]. Briefly, calibration standards were prepared by spiking the DPTIP into naïve plasma from mice and dogs. All the calibration curves were constructed in the range of 1–3000 pmol/mL in the naïve sample-matched matrices in both the mouse and dog samples. Linear regression with 1/(nominal concentration) weighting factor was used for fitting the calibration curve. A correlation coefficient greater than 0.99 was considered acceptable in the analytical runs for all analyses.

The plasma samples from both mice and dogs were processed through simple protein precipitation. For this, 20 µL of plasma was mixed with 100 µL methanol containing 0.5 µM losartan (internal standards; IS) in low retention microcentrifuge tubes. All samples were vortex-mixed and centrifuged at 16,000× *g* for 5 min at 4 °C. A 50 µL of aliquot of supernatant was diluted with 50 µL of LC-MS water transferred to a 96-well polypropylene autosampler sealed with a Teflon cap. A volume of 2 µL was injected onto the ultra-performance liquid chromatography instrument for quantitative analysis using a temperature-controlled autosampler operating at 4 °C. Chromatographic separation was performed using a Thermo Scientific Vanquish ultra-high-performance system consisting of an analytical pump and coupled with a TSQ Altis mass spectrometer. The analytes were separated using an Agilent Eclipse Plus column (100 × 2.1 mm i.d, 1.8 μm) maintained at 35 °C. The (M+H)^+^ ion transition of DPTIP (*m*/*z* 378.85 → 363.07, 200.05) and losartan (IS) (*m/z* 423.2 → 207.10, 180.88) were used. A gradient method was used with a mobile phase composition of 0.1% formic acid in acetonitrile and 0.1% formic acid in water.

### 2.7. Pharmacokinetic Analysis

The plasma concentrations (pmol/mL) for the mice were quantified, and plots of mean plasma concentration versus time were constructed using GraphPad Prism (10.3.0). Similarly, plasma concentrations (pmol/mL) for the dogs were measured and corresponding mean plasma concentration versus time plots were generated. The plasma concentrations in both mice and dogs were analyzed using a noncompartmental analysis method as implemented in the computer software program Phoenix WinNonlin version 8.4 (Certara USA Inc., Princeton, NJ, USA). The maximum plasma concentration (C_max_) and time to C_max_ (T_max_) were the observed values. The area under the plasma concentration–time curve (AUC) values were calculated to the last quantifiable sample (AUC_0–t_) using the log-linear trapezoidal rule. The oral bioavailability was calculated by AUC_PO_ divided by AUC _IV_ with dose normalization. DPTIP exposures in the mice were analyzed using Bailer’s method with a Bonferroni correction applied for multiple comparisons [48,49].

## 3. Results and Discussion

### 3.1. Chemistry

DPTIP prodrugs were synthesized by modifying the phenolic hydroxyl group with various promoieties, including simple alkyl esters, carbamates, piperidinyl carbamates, and phosphate esters. We previously reported single time point plasma and brain levels of DPTIP in the mice [36]. Of these prodrug **7** (containing diethyl piperidinopiperidyl moiety) was evaluated in a time-dependent pharmacokinetic studyand demonstrated over a 4-fold increase in DPTIP plasma exposure (AUC_0–t_ = 1047 vs. 270 pmol.h/mL) compared to DPTIP [36]. In this report, we selected previous prodrugs that showed a similar or higher release of DPTIP in mice and conducted full time course PK studies in mice and dogs to make a head-to-head comparison in oral exposures.

**Scheme 1:**
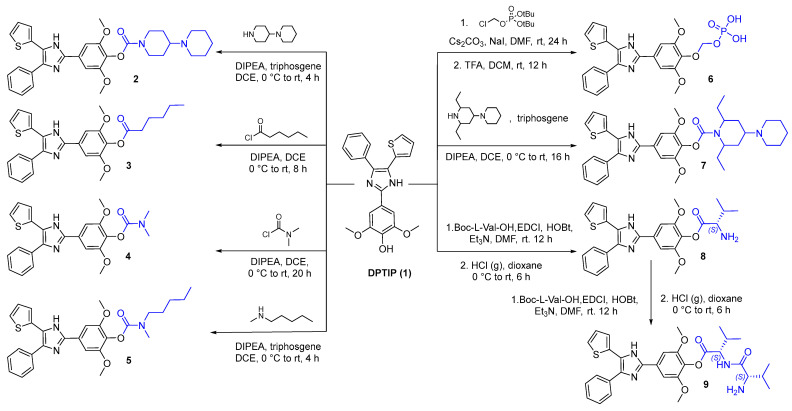
Schematic illustration of synthesis of prodrugs **2**–**9** from DPTIP using various carbamates, ester, and alkyloxy phosphate promoieties. Reagents: N,N-Diisopropylethylamine (DIPEA), 1,2-Dichloroethane (DCE), Trifluoroacetic acid (TFA), Dichloromethane (DCM), N,N-Dimethylformamide (DMF), 1-Ethyl-3-(3-dimethylaminopropyl) carbodiimide hydrochloride (EDCl), Hydroxybenzotriazole (HOBt), Triethylamine (Et_3_N), hydrogen chloride gas (HCl (g)), sodium iodide (NaI), cesium carbonate (Cs_2_CO_3_).

In addition to the previously synthesized prodrugs, which were intended for passive transport, we expanded the chemical space in this report by synthesizing 2 valine ester prodrugs (see Scheme 1) in an attempt to improve bioavailability by targeting intestinal transporters, as this strategy was successfully employed in other drugs. For instance, the valine ester prodrug of valacyclovir was shown to increase oral bioavailability by 3–5-fold by enhancing membrane transport via the peptide transporter PEPT1 in the intestine [50]. Similarly, the double valine ester prodrug of fluorodeoxyuridine, an anticancer drug, was shown to prevent intestinal activation, reducing severe intestinal toxicity [51,52]. The double valine ester prodrug of lopinavir also caused an increase in oral absorption by a demonstrated increased aqueous solubility, evasion of P-glycoprotein efflux, and prevention of CYP3A4-mediated metabolism [53,54].

The procedures used for the synthesis of new prodrugs **8**–**9** or scale up of previously published compounds **2**–**7** is described in Scheme 1. Briefly, DPTIP was prepared in three synthetic steps, starting with phenylacetylene using published procedures [55], and was used for the synthesis of prodrugs **2**–**7**, as we described [36]. New prodrugs **8** and **9** were prepared in 2–4 synthetic steps, respectively (Scheme 1).

### 3.2. In Vitro Plasma Stability of DPTIP and Its Prodrugs

DPTIP and its prodrugs **2**–**7** were evaluated for their stability in *CES1^−/−^* mice plasma previously [36]. To further investigate interspecies differences and gain an understanding of metabolic stability across species, we additionally assessed the stability of DPTIP (**1**) and its prodrugs (**2**–**9**) in dog and human plasma. The results, illustrated in Table 1, show that DPTIP and **2**, **4**, **6,** and **7** demonstrated high stability, with over 90% remaining intact after 60 min in all samples across species. Additionally, **5** and **9** showed moderate stability (40–77% remaining) in all tested species. Interestingly **3** (hexanoate ester prodrug) showed instability in mouse (<25%), moderate stability (~60%) in dog, and high stability (~80%) in human plasmas. Conversely, single valine prodrug **8** showed high instability across all tested species (~0% remaining).

Prodrugs **3**, **8**, and **9**, featuring ester promoieties, exhibited significant hydrolysis in mouse plasma, with less than 55% of the parent compound remaining at 60 min. In contrast, these prodrugs demonstrated greater stability in dog and human plasmas with 60–85% of the parent compound remaining. This difference is attributed to the lower expression of carboxylesterase enzymes in higher species such as dogs and humans, presumably leading to reduced ester hydrolysis [56]. Interestingly, single valine ester prodrug **8** was highly metabolized in plasma but the addition of double valine in **9** improved the stability across species, most likely due to steric hindrance of the diamino acids. This trend of greater instability for ester-based prodrugs in mice compared to dogs and humans can be attributed to the higher abundance of hydrolytic enzymes present in rodents relative to non-rodent species [56]. Prodrug **6**, with the methyl phosphonate moiety, was also stable in plasma as it was designed to be metabolized by alkaline phosphatases, which are highly abundant in the liver and gastrointestinal tract but not in plasma [57].

In contrast to esters, prodrugs with carbamate-linked piperidino-piperidinyl promoieties (**2** and **7**) were stable in the plasma due to the inherent stability of carbamates [58]. However, an exception was **5,** which, despite its carbamate group, showed moderate stability across all species. This reduced stability is attributed to a longer alkyl chain, which increases susceptibility to hydrolysis than its cyclic carbamate counterparts [59].

### 3.3. Ester and Phosphonate-Based Prodrugs Showed Improved Plasma Concentration in Mice

Prodrugs **2** (piperidinopiperidinyl promoiety) and **7** (diethyl piperidinopiperidinyl promoiety) were shown to improve the PK properties of DPTIP in mice following oral administration [36]. Prodrug **2** demonstrated similar DPTIP plasma exposure (273 vs. 270 pmol·h/mL) but enhanced brain exposure. In contrast, prodrug **7** achieved a remarkable 4-fold higher plasma exposure of DPTIP compared to the free drug (1047 pmol·h/mL vs. 270 pmol·h/mL). Previously reported PK data for DPTIP and prodrugs **2** and **7** in mice were used as benchmarks to assess improvements with other prodrugs in this study.

Specifically, we selected previously reported prodrugs **3** (hexanoyl ester), **4** (dimethyl carbamate), **5** (pentyl carbamate), and **6** (methyl phosphonate) that showed either a similar or higher release of DPTIP in plasma [36]. Additionally, the newly designed valine ester prodrugs **8** and **9** were evaluated for PK studies. All prodrug studies were conducted in CES1^−/−^ mice (n = 3) at an equivalent dose of 10 mg/kg DPTIP.

**Figure 1:**
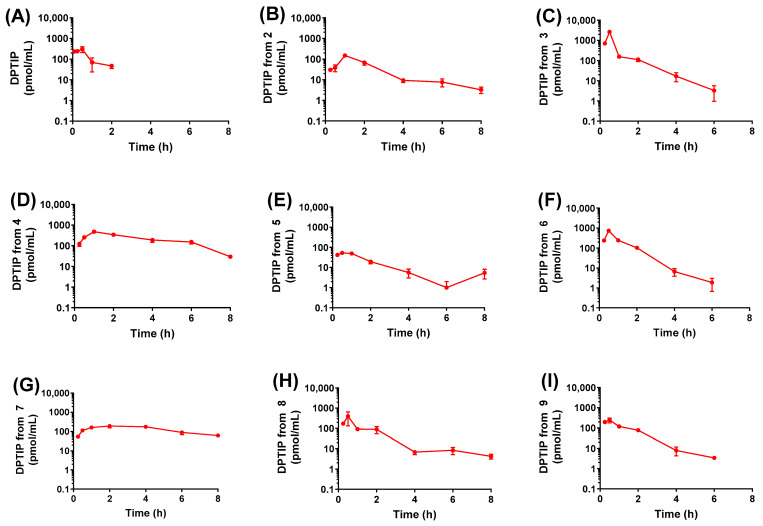
Plasma concentration–time profiles of DPTIP and released DPTIP from its prodrugs in *CES1^−/−^* mice (**A**) DPTIP * (**B**) 2*, (**C**) **3**, (**D**) **4**, (**E**) **5**, (**F**) **6**, (**G**) 7*, (**H**) **8,** and (**I**) **9**. Graphs show DPTIP profile in mice following single oral dose of DPTIP (10 mg/kg) or its prodrugs (10 mg/kg equivalent of DPTIP) with plasma collected at predetermined time points. Data expressed as mean ± SEM (*n* = 3 mouse/time point). * Previously reported [36].

Prodrug **4** demonstrated the highest DPTIP plasma exposure with a ~6-fold increase in AUC_0–t_, while prodrugs **3** and **6** also showed significant increases of 5.5-fold and 2.3-fold, respectively (Figure 1, Table 2). In contrast, prodrug **5** exhibited decreased plasma exposure (AUC_0–t_ = 117 vs. 270 pmol·h/mL) and was excluded from further evaluation. Surprisingly, prodrugs **8** (single valine ester) and **9** (double valine ester) yielded only marginal increases in plasma exposure compared to DPTIP, with **8** achieving a 1.3-fold increase (AUC_0–t_ = 368 vs. 270 pmol·h/mL) and **9** achieving a 1.2-fold increase (AUC_0–t_ = 316 vs. 270 pmol·h/mL) (Figure 1, Table 2). Despite their marked differences in plasma stability, the double valine ester prodrug **9** was likely rapidly converted during pre-absorption in the gastrointestinal tract of mice not yielding substantial differences in exposures versus DPTIP.

A major limitation of DPTIP is its short half-life (t_1/2_ = 0.46 h), which poses challenges for efficacy testing in mouse models. Interestingly, the use of prodrug strategies resulted in a substantial extension of DPTIP’s half-life, achieving improvements ranging from 1.3- to 6-fold. Most prodrugs demonstrated more than a 2-fold increase in half-life, with the exception of prodrugs **3** and **6**, which showed no significant improvement.

In summary, amongst the 8 tested prodrugs in mice, only compound **5** exhibited low plasma exposure levels and was therefore excluded from further PK studies in dogs. Prodrugs **3**, **4**, **6**, and **7** demonstrated significantly a higher exposure of DPTIP compared to DPTIP administration, as determined using Bailer’s method [48,49]. In contrast, prodrugs **2**, **8**, and **9** showed similar or higher plasma exposures, which were not statistically significant. All prodrugs, excluding **5,** improved half-lives in mice, indicating better PK profiles. As a result, these compounds (with the exception of **5**) were selected for additional PK evaluation in dogs to further assess their potential for clinical development.

### 3.4. Double Valine Ester-Based Prodrug **9** Showed 2-Fold Increase in Oral Bioavailability Compared to DPTIP

**Figure 2:**
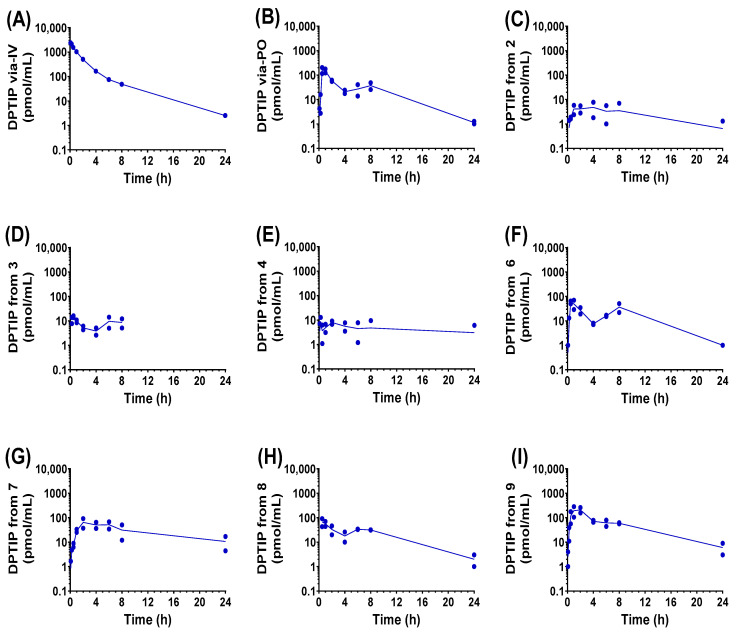
Plasma concentration–time profiles of DPTIP and released DPTIP from its prodrugs in dogs (**A**) DPTIP-IV, (**B**) DPTIP-PO (**C**) **2**, (**D**) **3**, (**E**) **4**, (**F**) **6**, (**G**) **7**, (**H**) **8**, and (**I**) **9**. DPTIP administered via IV (1 mg/kg) or PO (2 mg/kg) with prodrugs (2 mg/kg DPTIP equivalent) and plasma collected at predetermined time points. Plasma samples analyzed using our developed LC/MS-MS methods.

DPTIP administered intravenously (IV) at a dose of 1 mg/kg in dogs achieved a maximum plasma concentration (C_max_) of 2.4 nmol/mL and exposures (AUC_0–t_) of 3.9 nmol·h/mL (Figure 2 and Table 3). The initial plasma concentration (C_0_) was determined to be 2.5 nmol/mL, and the volume of distribution (V_d_) was calculated at 3.8 L/kg, indicating a high distribution of DPTIP. The clearance (CL) of DPTIP was moderate at 12 mL/min/kg, and the half-life was calculated to be 3.7 h.

For the oral PK studies, a dose of 2 mg/kg of DPTIP or its prodrugs (equivalent to DPTIP) was selected based on their solubility in formulations suitable for dog studies. Following oral administration, DPTIP achieved an AUC_0–t_ of 701 pmol·h/mL and an oral bioavailability of 8.97% (Table 3). Notably, its half-life was significantly prolonged in dogs (3.7 h) compared to mice (0.46 h). This difference may be attributed to species-specific clearance mechanisms, potentially involving rapid renal clearance mediated by phase II glucuronidation of the phenolic hydroxyl in mice [35].

The exposures of DPTIP from prodrugs are given in Table 3. Most of the tested prodrugs displayed lower exposure (AUC_0–t_) compared to DPTIP, corresponding to decreased oral bioavailability (≤9%) (Figure 2 and Table 3). Unfortunately, prodrug **7**, previously identified as the best prodrug in mice [36], failed to enhance DPTIP exposure in dogs. Its levels were comparable to that of DPTIP (693 vs. 701 pmol·h/mL), resulting in a similar oral bioavailability (8.87%). This outcome may be attributed to impaired absorption, insufficient metabolic activation, or increased biliary excretion in dogs.

Interestingly, newly designed valine ester prodrug **9** demonstrated enhanced PK properties with a 1.2-fold increase in C_max_ (219 pmol/mL) and a remarkable ~2-fold improvement in exposures (AUC_0–t_ of 1352 pmol.h/mL vs. DPTIP = 701 pmol.h/mL). This led to the enhancement of oral bioavailability to 17.3% (Figure 2 and Table 3). Notably of the tested prodrugs, **9** with its double valine moiety remains the only compound that showed improvement in plasma exposure in both mouse and dog PK.

While overall exposures were not significantly enhanced by the prodrugs, similar to mice, most of the prodrugs improved the half-life of DPTIP by 1.1–2.4-fold in dogs, with the exception of prodrug **3**. Carbamate-based prodrugs **2**, **4**, and **7**, which exhibited the longest half-life in mice (>3.5-fold), also showed a similar trend in dogs (>1.5-fold). The single and double valine ester prodrugs **8** and **9** demonstrated a modest 1.1–1.4-fold increase in half-life. Despite these improvements however most prodrugs exhibited poor pharmacokinetic properties in dogs compared to mice. Interestingly, DPTIP itself displayed superior pharmacokinetics in dogs, with a markedly prolonged half-life of 3.7 h compared to 0.46 h in mice, potentially due to interspecies variations in phase II glucuronidation of the phenolic hydroxyl group.

Although detailed mechanistic studies to investigate these differences were not feasible in larger species such as dogs, these disparities were attributed to well-documented interspecies differences in the ADME (absorption, distribution, metabolism, and excretion) of small molecules. One key factor is the considerable differences in gastrointestinal (GI) physiology across species. For instance, the intestinal pH in dogs can range from 3 to 8, which can markedly influence the dissolution and solubility of prodrugs, thereby affecting their absorption and overall PK profile [60]. Additionally, interspecies differences in biliary excretion can also influence the bioavailability of prodrugs between mice and dogs. In general, the extent of biliary excretion is known to be much higher in dogs and rats compared to other species [61,62]. Furthermore, when combined with our metabolic stability data, the slower pre-hepatic hydrolysis rate observed in dogs could result in a greater proportion of biliary clearance of intact compounds [63].

This study, to the best of our knowledge, represents one of the most comprehensive pharmacokinetic evaluations of nSMase2 inhibitors, with emphasis on DPTIP. Notably, the PKs of other effective nSMase2 inhibitors, such as GW4869 and Cambinol, has not been reported, likely due to their low potency and poor physicochemical properties [32,33]. While the PKs of PDDC, a compound discovered by our group, was reported, it is about 10-fold less potent than DPTIP [64]. Therefore, our focus in this study was on comparing DPTIP (the most potent compound identified to date) to its prodrugs, which demonstrated superior exposure and half-life in two species. This study also highlights the importance of considering species-specific factors in prodrug development, which is essential for successful clinical translation [38,65,66]. Prodrug **9**, with its extended half-life and significantly improved exposure in both mice and dogs, emerges as a promising lead for further pharmacological evaluation and potential clinical translation.

## 4. Conclusions

DPTIP prodrugs from diverse categories, including simple alkyl esters, carbamates, piperidinyl carbamates, phosphate esters, and amino acid esters, were selected to investigate interspecies PK differences in mice and dogs. Among these, only the double valine ester prodrug **9** demonstrated notable improvements in PK parameters compared to DPTIP. This study highlights the challenges of translating PK results from small animal models to non-rodent species like dogs. Encouragingly, ongoing efforts to optimize prodrugs based on **9** are focused on enhancing oral bioavailability, with the ultimate goal of advancing these candidates for clinical applications targeting EVs and nSMase2 inhibition.

## Figures and Tables

**Table 1:** In vitro stability of DPTIP and its prodrugs (**2**–**9**) in mouse, dog, and human plasmas.

Compound	R	In Vitro Stability in Plasma (% Remaining at 60 min) Mean ± SEM (n = 3)		
		Mice	Dogs	Humans
**DPTIP**	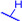	103 ± 1.9 *	100 ± 2.3	100 ± 5.0
**2**	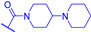	89.7 ± 2.5 *	101± 2.7	89.9 ± 2.5
**3**	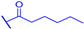	13.8 ± 1.7 *	62.9 ± 7.2	79.3 ± 1.4
**4**	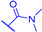	96.1 ± 6.5 *	104 ± 7.9	101 ± 3.2
**5**	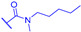	59.9 ± 5.8 *	49.5 ± 1.0	42.3 ± 2.1
**6**	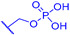	96.2 ± 8.2 *	108 ± 5.2	93.3 ± 0.6
**7**	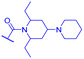	91.6 ± 4.1 *	94.7 ± 3.4	96.8 ± 1.8
**8**	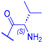	0 ± 0	0 ± 0	0 ± 0
**9**	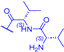	53.2 ± 5.1	76.3 ± 1.8	76.4 ± 1.4

* Previously published [36].

**Table 2:** In vivo pharmacokinetic parameters of released DPTIP from DPTIP or its prodrugs in mice dosed orally at 10 mg/kg or equimolar dose.

Compound	Dose (mg/kg)	C_max_ (pmol/mL)	T_max_ (h)	AUC_0–t_ (pmol·h/mL)	t _(1/2)_ (h)
**DPTIP** *	10	307 ± 93.9	0.25	270 ± 64.3	0.46
**2** *	10 ^#^	149 ± 9.9	1	273 ± 36.8	1.4
**3**	10 ^#^	2636 ± 225	0.5	1491 ± 51.5 ^α^	0.59
**4**	10 ^#^	485 ± 52.3	1	1667 ± 145 ^α^	2.0
**5**	10 ^#^	56 ± 8.4	0.5	116 ± 16.6	0.98
**6**	10 ^#^	737 ± 60.5	1	616 ± 27.6 ^α^	0.62
**7** *	10 ^#^	191 ± 39.6	2	1047 ± 101 ^α^	2.7
**8**	10 ^#^	412 ± 248	0.5	368 ± 122	1.39
**9**	10 ^#^	249 ± 62.7	0.25	316 ± 67.3	0.81

* Previously published [36]. ^#^ Dose equivalent to DPTIP. ^α^ *p* < 0.05 statistically significant compared to DPTIP by Bailer analysis with Bonferroni correction.

**Table 3:** In vivo pharmacokinetic parameters of released DPTIP from DPTIP or its prodrugs in dogs.

Compound	Dose (mg/kg)	Route	C_max_ (pmol/mL)	T_max_ (h)	AUC_0–t_ (pmol·h/mL)	t_1/2_ (h)	% F
**DPTIP**	1	IV	4796 *	NA	7809 *	3.71	NA
**DPTIP**	2	PO	190	0.5	701	3.74	8.97
**2**	2 ^#^	PO	5.3	2	68.6	5.2	0.87
**3**	2 ^#^	PO	15.2	0.5	127	1.96	1.62
**4**	2 ^#^	PO	11.2	2	106	9	1.35
**6**	2 ^#^	PO	60.0	0.5	484	3.88	6.19
**7**	2 ^#^	PO	64.4	2	693	8.12	8.87
**8**	2 ^#^	PO	68.0	1	520	4.19	6.66
**9**	2 ^#^	PO	219	1	1352	5.41	17.31

* Dose adjusted to 2 mg/kg, ^#^ Dose equivalent to DPTIP.

## Data Availability

All data are contained within the article.

## Supplementary Materials

The following supporting information can be downloaded at: https://www.mdpi.com/article/10.3390/pharmaceutics17010020/s1, Table S1. Molecular weight, ionization mode and its [M + H] ^+^ obtained in the full scan mode (from m/z 75.00 to 1125.00) of DPTIP and its prodrugs (**2**–**9**) in the LC/MS analysis; Figure S1. (A) ^1^H NMR spectrum of **8** in DMSO-*d*_6_ (B) LC-MS spectrum of **8** (retention time: 4.28 min; purity: >99%); Figure S2. (A) ^1^H NMR spectrum of **9** in DMSO-*d*_6_ (B) LC-MS spectrum of **9** (retention time: 4.38 min; purity: >97%).
