# Supplementary material for: Pharmacokinetic Evaluation of Neutral Sphinghomyelinase2 (nSMase2) Inhibitor Prodrugs in Mice and Dogs
Source: Pharmaceutics. 2024 Dec 26;17(1):20. doi: 10.3390/pharmaceutics17010020 (PMC11768932; doi:10.3390/pharmaceutics17010020)
Supplement: Supplementary file 1 [file pharmaceutics-17-00020-s001.zip › pharmaceutics-3390173-supplementary.pdf]

Pharmacokinetic Evaluation of Neutral Sphinghomyelinase2 (nSMase2) Inhibitor Prodrugs in Mice and Dogs

Arina Ranjit [1,2], Chae Bin Lee [1,2], Lukáš Tenora [1,2], Vijaya Saradhi Mettu [1,2], Arindom Pal [1,2], Jesse Alt [1,2], Barbara S. Slusher [1,2,3,4,5,6,7,*] and Rana Rais [1,2,4,*]

1 Department of Neurology, Johns Hopkins School of Medicine, Baltimore, MD 21205, USA
2 Johns Hopkins Drug Discovery, Johns Hopkins School of Medicine, Baltimore, MD 21205, USA
3 Departments of Psychiatry and Behavioral Sciences, Johns Hopkins School of Medicine, Baltimore, MD 21205, USA
4 Department of Pharmacology and Molecular Sciences, Johns Hopkins School of Medicine, Baltimore, MD 21205, USA
5 Department of Oncology, Johns Hopkins School of Medicine, Baltimore, MD 21205, USA
6 Department of Neuroscience, Johns Hopkins School of Medicine, Baltimore, MD 21205, USA
7 Department of Medicine, Johns Hopkins School of Medicine, Baltimore, MD 21205, USA
* Correspondence: bslusher@jhmi.edu (B.S.S.); rrais2@jhmi.edu (R.R.)

Table S1. Molecular weight, ionization mode and its [M + H] + obtained in the full scan mode (from m/z 75.00 to 1125.00) of DPTIP and its prodrugs (**2**-**9**) in the LC/MS analysis.

**Compound**	**Mol wt g/mol**	**Ionization mode**	**Q1 [M + H] +**
**DPTIP**	378.104	ESI, MRM (+)	379.1110
**2**	572.246	ESI, MRM (+)	573.2529
**3**	476.177	ESI, MRM (+)	477.1842
**4**	449.141	ESI, MRM (+)	450.1481
**5**	491.188	ESI, MRM (+)	492.195
**6**	488.081	ESI, MRM (+)	489.0879
**7**	628.308	ESI, MRM (+)	629.3155
**8**	477.172	ESI, MRM (+)	478.1794
**9**	576.241	ESI, MRM (+)	577.2479

Chemistry

Synthesis of Prodrug 8

*2,6-Dimethoxy-4-(4-phenyl-5-(thiophen-2-yl)-1H-imidazol-2-yl) phenyl (tert-butoxycarbonyl)-L-valinate (**8a**).*

DPTIP (1.00 g, 2.64 mmol, 1 equivalent.) was dissolved in anhydrous DMF (15 ml) and (tert-butoxycarbonyl)-L-valine (860 mg, 3.96 mmol, 1.5 equivalent.), 1-ethyl-3-(3-dimethylaminopropyl) carbodiimide hydrochloride (750 mg, 3.96 mmol, 1.5 equivalent.), 1-hydroxybenzotriazole (530 mg, 3.96 mmol, 1.5 equivalent.) and triethyla-mine (1.1 mL, 7.92 mmol, 3 equivalent.) were added and the resulting mixture was stirred at room temperature for 12 h. The reaction mixture was diluted with EtOAc (50 mL), washed with dist. water (2×10 mL) and sat. NaCl (10 mL). Organic layer was sepa-rated, dried over anhydrous Na2SO4 and volatiles were removed under reduced pres-sure. The residue was purified by LC on silica (EtOAc/PE, 4:1) to give **8a** (800 mg) as an off-white solid in 80% yield.

LC-MS: 578.0 (M+H$^+$).

2,6-Dimethoxy-4-(4-phenyl-5-(thiophen-2-yl)-1H-imidazol-2-yl) phenyl L-valinate hy-drochloride (8).

Compound **8a** (800 mg, 2.64 mmol, 1 equivalent) was dissolved in anhydrous 1,4-dioxane (10 mL) and 4 M hydrochloric acid in 1,4-dioxane (5 mL) was dropwise added at 0 °C. The resulting mixture was slowly heated to room temperature and stirred for further 6 h. The volatiles were removed under reduced pressure, the residue was triturated with diethyl ether, filtrated and dried under high vacuo to give the title **8** (520 mg, hydrochloric salt) as an off-white solid in 65% yield.

^{1}H-NMR (400 MHz, DMSO-*d6*) δ 8.80 – 7.62 (m, 3H), 7.95 (brs, 2H), 7.81 (brs, 1H), 7.66 (brs, 3H), 7.57 (brs, 1H), 7.50 – 7.41 (m, 3H), 7.12 (brs, 1H), 4.42 – 4.33 (m, 1H), 3.89 (s, 6H), 2.40 – 2.32 (m, 1H), 1.05 (d, J = 4.0 Hz, 6H)

LC-MS: 477.9 (M +H$^+$).

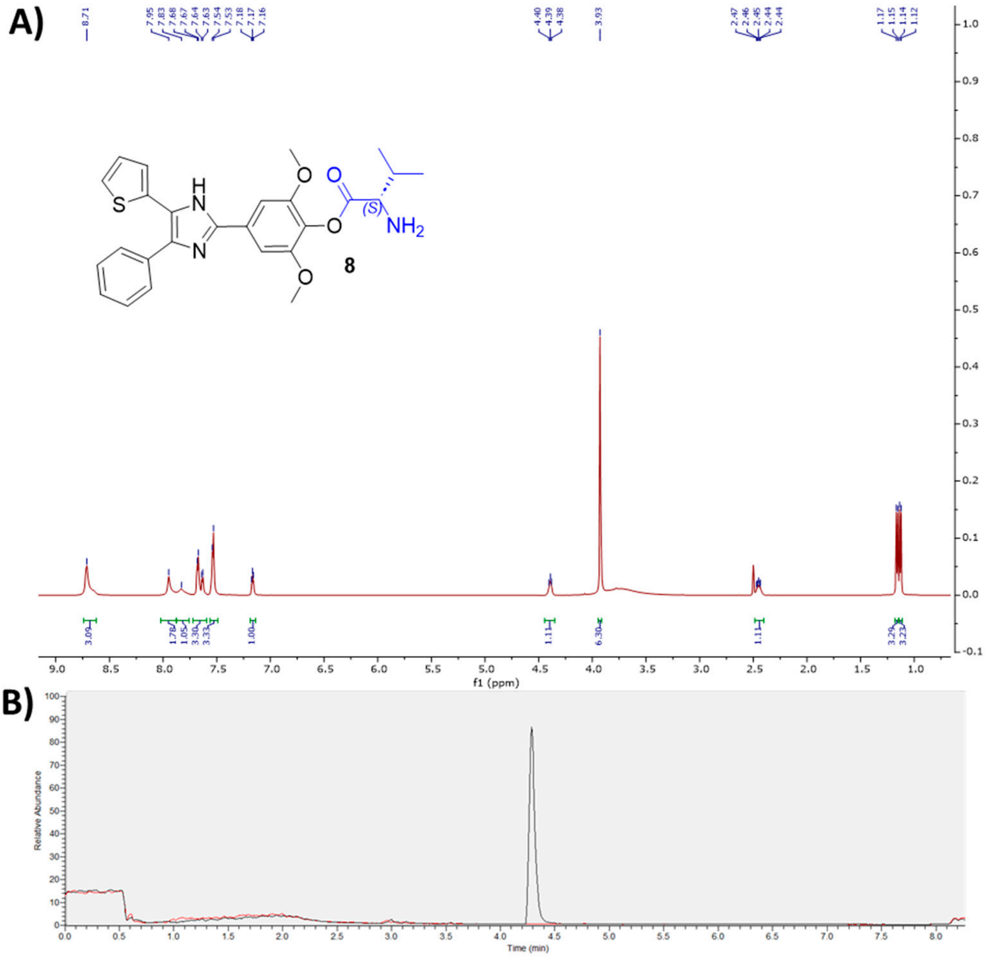

Figure S1. (A) ^1H NMR spectrum of **8** in DMSO-d_6 (B) LC-MS spectrum of **8** (retention time: 4.28 min; purity: >99%).

Synthesis of Prodrug 9

*2,6-Dimethoxy-4-(4-phenyl-5-(thiophen-2-yl)-1H-imidazol-2-yl) phenyl (tert-butoxycarbonyl)-L-valyl-L-valinate (**9a**).*

Compound **8** (520 mg, 1.10 mmol, 1 equivalent.) was dissolved in anhydrous DMF (10 ml) and (tert-butoxycarbonyl)-L-valine (320 mg, 1.51 mmol, 1.5 equivalent.), 1-ethyl-3-(3-dimethylaminopropyl) carbodiimide hydrochloride (290 mg, 1.51 mmol, 1.5 equivalent.), 1-hydroxybenzotriazole (200 mg, 1.51 mmol, 1.5 equivalent.) and tri-ethyalmine (420 µL, 3.02 mmol, 3 equivalent.) were added and the resulting mixture was stirred at room temperature for 12 h. The reaction mixture was diluted with EtOAc (20 mL), washed with dist. water (2×10 mL) and sat. NaCl (10 mL). Organic layer was sepa-rated, dried over anhydrous Na2SO4 and volatiles were removed under reduced pres-sure. The residue was purified by LC on silica (EtOAc/PE, 4:1) to give 9a (370 mg) as an off-white solid in 71% yield.

LC-MS: 676.9 (M+H$^+$).

*2,6-Dimethoxy-4-(4-phenyl-5-(thiophen-2-yl)-1H-imidazol-2-yl) phenyl L-valyl-L-valinate hydrochloride (**9**).*

Compound **9a** (370 mg, 0.060 mmol, 1 equivalent) was dissolved in anhydrous 1,4-dioxane (5 mL) and 4 M hydrochloric acid in 1,4-dioxane (4 mL) was dropwise added at 0 °C. The resulting mixture was slowly heated to room temperature and stirred for further 6 h. The volatiles were removed under reduced pressure, the residue was triturated with diethyl ether, filtrated and dried under high vacuo to give the title **9** (150 mg, hydrochloric salt) as an off-white solid in 40% yield.

^{1}H-NMR (400 MHz, DMSO-d6) δ 8.32 (d, J = 8.0 Hz, 1H), 8.25 (brs, 3H), 7.76 (brs, 2H), 7.66 – 7.63 (m, 4H), 7.52 (brs, 3H), 7.16 (s, 1H), 4.61 – 4.60 (m, 1H), 3.87 (s, 6H), 3.81 – 3.80 (m, 1H), 2.33 – 2.30 (m, 1H), 2.22 – 2.20 (m, 1H), 1.09 (d, J = 4.0 Hz, 6H), 0.98 (d, J = 4.0 Hz, 6H). LC-MS: 577.1 ($M+H^+$).

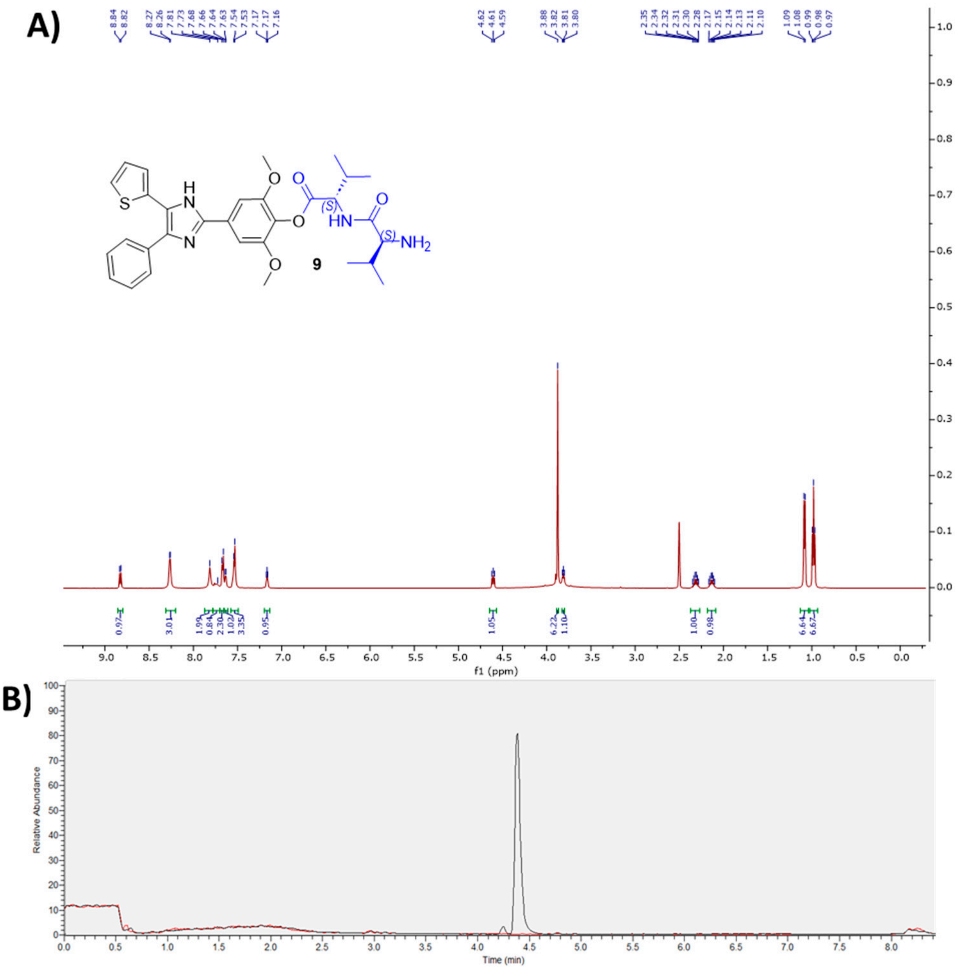

Figure S2. (A) ^{1}H NMR spectrum of **9** in DMSO-d_6 (B) LC-MS spectrum of **9** (retention time: 4.38 min; purity: >97%).

Conditions for LC-MS analysis

We utilized Dionex ultra-high-performance LC system coupled with a Q Exactive Focus orbitrap mass spectrometer (Thermo Fisher Scientific Inc., Waltham MA). The analysis was performed in the full scan mode (from m/z 75 to 1125). The analytes were separated using an Agilent Eclipse Plus C_{18} column (100 × 2.1 mm i.d, 1.8 µm) maintained at 35 °C. A gradient method was used with mobile phase of composition of 0.1% formic acid in acetonitrile and 0.1% formic acid in water.
